# Efficacy and Safety of Shen-Ling-Lian-Xia Granule Combined With Neoadjuvant Chemotherapy in Patients With Triple-Negative Breast Cancer: Protocol for a Randomized, Double-Blind, Multicenter Clinical Trial

**DOI:** 10.2196/91475

**Published:** 2026-05-11

**Authors:** Yuanyuan Ren, Shuai Yuan, Youyang Shi, Lichen Tang, Tian Li, Rui Yang, Kaigang Xie, Xiaoqing Zhang, Jue Chen, Jinhui Hu, Sheng Liu

**Affiliations:** 1 School of Clinical Medicine, Jiangxi University of Chinese Medicine, Jiangxi, China; 2 Department of Breast Surgery, Longhua Hospital, Shanghai University of Traditional Chinese Medicine, 725 Wanping South Rd, Xuhui District, Shanghai, 200032, China, 86 18917763005; 3 International Peace Maternity & Child Health Hospital, Shanghai Jiao Tong University School of Medicine, Shanghai, China; 4 Fudan University Shanghai Cancer Center, Shanghai, China; 5 Shanghai Baoshan Hospital of Integrated Traditional Chinese and Western Medicine (Baoshan Hospital, Shanghai University of Traditional Chinese Medicine), Shanghai, China; 6 Shanxi Cancer Hospital, Taiyuan, China; 7 Ningbo Hospital of Integrated Traditional Chinese and Western Medicine, Ningbo, China; 8 Jiangsu Province Hospital of Traditional Chinese Medicine, Jiangsu, China; 9 Affiliated Hospital of Yangzhou University, Yangzhou, China; 10 First Affiliated Hospital of Hunan University of Chinese Medicine, Hunan, China

**Keywords:** triple-negative breast cancer, neoadjuvant chemotherapy, Shen-Ling-Lian-xia Granules, randomized controlled trial, integrative medicine

## Abstract

**Background:**

Neoadjuvant chemotherapy (NAC) is a crucial component of systemic treatment for triple-negative breast cancer (TNBC), serving as an effective means to reduce recurrence rates and improve survival outcomes. It not only maximizes the extent of resectable tumors but also converts inoperable tumors into operable ones. Shen-Ling-Lian-Xia Granules (SLLXG) is an in-house preparation developed by Longhua Hospital, affiliated with Shanghai University of Traditional Chinese Medicine. Formulated by Professor Liu Sheng, the fifth-generation inheritor of the Shanghai Gu School of Surgery, based on extensive clinical experience treating breast cancer, this compound has been clinically proven to improve postoperative symptoms in patients with TNBC, enhance immunity, reduce recurrence and metastasis, and prolong disease-free survival. However, clinical evidence regarding its efficacy in enhancing the therapeutic effect of NAC for TNBC remains to be established.

**Objective:**

This study aims to analyze the impact of SLLXG on the efficacy of NAC for TNBC, evaluate its synergistic effect on NAC, and identify potential beneficiary populations for SLLXG in treating TNBC. It seeks to establish a standardized diagnostic and treatment protocol for broader clinical implementation.

**Methods:**

This study will recruit 306 patients diagnosed with TNBC. Patients will be randomly assigned to two groups: one receiving NAC combined with SLLXG treatment, and the other receiving NAC combined with SLLXG placebo treatment. The primary efficacy endpoint was pathological complete response. Secondary efficacy endpoints included objective response rate, clinical benefit rate, Miller-Payne grading for breast cancer, residual cancer burden, peripheral blood lymphocyte levels, stromal tumor-infiltrating lymphocytes in the tumor region stroma, and European Organisation for Research and Treatment of Cancer Quality of Life Questionnaire (EORTC QLQ-C30; version 3.0). Evaluation timepoints will occur after completion of cycles 2, 4, 6, and 8 of NAC. Data will be analyzed using SPSS (version 25.0; IBM Corp) to compare within-group and between-group differences between the 2 cohorts, with a significance level of α=.05 for hypothesis testing.

**Results:**

The study was funded in January 2025 and was approved by the Medical Ethics Committee in October 2025. Patient recruitment is scheduled to commence in February 2026. As of January 2026, no patients have been enrolled. The study is expected to conclude data collection by December 2027, with data analysis and final results anticipated for publication in spring 2028.

**Conclusions:**

The findings of this study will validate the efficacy of SLLXG in treating TNBC and confirm its synergistic effect on treatment outcomes compared to NAC alone.

## Introduction

### Background

Breast cancer remains the most diagnosed malignancy and a leading cause of cancer-related mortality among women globally. According to the Global Burden of Disease Study 2023, the incidence of breast cancer is projected to continue rising through 2050, representing an escalating global health challenge [1]. Furthermore, recent comprehensive evidence spanning from 1990 to 2021 highlights that this persistent increase in age-standardized incidence rates is particularly pronounced in regions with a high sociodemographic index, underscoring the urgent need for optimized therapeutic strategies [2]. Triple-negative breast cancer (TNBC) accounts for 12% to 17% of all breast cancers. Due to its high recurrence rate and low survival rate, it is classified as a difficult-to-treat breast cancer [3]. Tumor size, number of lymph node metastases, and histological differentiation grade are critical factors influencing the treatment and prognosis of TNBC [4]. Neoadjuvant chemotherapy (NAC) is an effective strategy for reducing recurrence rates and improving survival in TNBC. It maximizes the extent of resection for operable tumors and can convert inoperable tumors into operable ones [5,6]. Pathological complete response (pCR) following NAC correlates with favorable prognosis in TNBC, including event-free survival and overall survival (OS) [7]. Early-stage TNBC should prioritize NAC as the core treatment strategy, with real-time stratification based on pCR rates to enable precision management [8]. Large-scale pooled analyses have firmly established pCR as a robust surrogate for long-term survival in this population, demonstrating hazard ratios of 0.24 for event-free survival and 0.16 for OS [9]. Despite the inherent chemosensitivity of TNBC, data from modern cohorts indicate that pCR rates remain approximately 37% [10]. This creates a critical prognostic divergence: patients achieving pCR enjoy an excellent 3-year OS of 94%, whereas those with residual disease experience a significantly worse prognosis, with a 3-year OS of only 68% and a high risk of early recurrence [11]. Consequently, novel strategies to further enhance pCR rates are urgently required.

Shen-Ling-Lian-Xia Granules (SLLXG) is an in-house preparation of Longhua Hospital affiliated with Shanghai University of Traditional Chinese Medicine. This formula was developed by Professor Liu Sheng, the fifth-generation inheritor of the Shanghai School of Gu’s Surgery, based on his extensive clinical experience in treating breast cancer. The formula comprises *Codonopsis, Curcuma, Atractylodes, Poria, Cnidium, Epimedium, Solanum nigrum, Scutellaria barbata*, and *Prunella vulgaris*. SLLXG was formerly known as Sanyin Fang (SYF). A multicenter prospective cohort study demonstrated that combining traditional Chinese medicine (TCM) with conventional therapy after breast surgery significantly accelerates the resolution of postoperative physical, sleep, depressive, and social functional symptoms in patients with TNBC, thereby enhancing health-related quality of life. The scales used in the study exhibited good reliability and validity [12]. A retrospective cohort study (n=348) demonstrated that the 24-month disease-free survival rate in the San Yin Fang intervention group was significantly higher than that in the nonintervention group (98.08% vs 89.06%; *P*<.05). Cox proportional hazards analysis confirmed that Chinese herbal medicine intervention was an independent protective factor (hazard ratio [HR] 0.191, 95% CI 0.083‐0.443), while metastatic lymph node stage was the sole risk factor. These findings suggest that the triple-negative formula effectively prolongs postoperative disease-free survival in patients with TNBC [13]. Previous multicenter work (n=252) showed that supplementing standard adjuvant chemotherapy with the traditional Chinese botanical compound SYF extends 5-year disease-free survival in operable TNBC (94.2% vs 85.5%; HR=0.40; *P*=.03) and confers an 8.7% absolute benefit, particularly among node-negative patients (HR=0.21), establishing SYF as a safe and promising botanical adjuvant for this population [14]. Furthermore, preliminary basic research using HPLC-MS/MS (high-performance liquid chromatography–tandem mass spectrometry) combined with network pharmacology and in vitro and in vivo models has, for the first time, elucidated that SYF targets the AKR1C3/MMPs/STAT3 axis through its active components (kaempferol, coumaric acid, vanillic acid, and 3,4-dihydroxyphenylacetic acid) to target the AKR1C3/MMPs/STAT3 axis, inhibit JAK/STAT3 signaling, and downregulate MMP-2/MMP-9. This mechanism blocks TNBC cell proliferation, migration, invasion, and in vivo metastasis, providing a mechanistic basis for the continued clinical development of SYF [15]. Crucially, the aberrant activation of the JAK2/STAT3 signaling pathway has been identified as a definitive pathological hallmark of TNBC, serving as a central hub that integrates multiple oncogenic signals [16]. Selective chemical probe inhibitors targeting the STAT3 SH2 domain (eg, S3I-201) have been shown to directly suppress the transcription of core survival factors, providing early preclinical evidence that STAT3 inhibition can trigger apoptosis in malignant breast cells [17]. Recent evidence highlights that STAT3 does not act in isolation; its interaction with other oncogenic transcription factors, such as GLI1, significantly amplifies the aggressive phenotype of TNBC cells, promoting enhanced proliferation and survival under therapeutic stress [18]. Advanced phosphorylation inhibitors like FLLL32 demonstrate that targeting this axis can significantly downregulate anti-apoptotic proteins, including Bcl-2 and Survivin, which are key drivers of therapeutic evasion in TNBC [19]. Beyond driving tumor growth, hyperactivated STAT3 is a master regulator of the immunosuppressive tumor microenvironment in TNBC [20]. It facilitates immune evasion by suppressing the activity of effector T cells while simultaneously inducing the expression of immune checkpoint molecules and inhibitory cytokines, thereby shielding tumor cells from antitumor immune responses [16,20]. Regulatory mechanisms, such as those mediated by miR-204, have shown that silencing the JAK2/STAT3 pathway not only facilitates immune recognition but also lowers the apoptotic threshold, thereby synergistically enhancing the sensitivity of cancer cells to conventional chemotherapeutic agents [21]. Given that SLLXG has demonstrated potent inhibitory effects on this specific axis [15], it represents a promising therapeutic strategy to counteract these pathological mechanisms and enhance the efficacy of NAC.

While previous multicenter studies have demonstrated the clinical efficacy of SYF/SLLXG in the adjuvant setting (postsurgery) [12-14], high-quality evidence regarding its synergistic benefits when integrated with NAC for TNBC remains insufficient. Given that TNBC is characterized by an immunosuppressive microenvironment and hyperactivation of oncogenic pathways such as JAK/STAT3 [16,18,20], and SLLXG has been shown to effectively regulate the tumor microenvironment and inhibit the JAK/STAT3 axis [15], we hypothesize that the concurrent administration of SLLXG during NAC will significantly enhance pCR rate, the primary endpoint of this study, by remodeling the immune landscape and reversing chemoresistance. To evaluate this hypothesis of superiority, the pCR rate will be compared between the intervention and control groups. Furthermore, we expect this combination therapy to improve secondary clinical outcomes, including objective response rate (ORR) and clinical benefit rate (CBR), while concurrently promoting favorable changes in immune biomarkers such as stromal tumor-infiltrating lymphocytes (sTILs). Ultimately, this study aims to provide a comprehensive validation of the synergistic clinical and biological efficacy of SLLXG in the neoadjuvant management of TNBC.

### Objectives

Building upon prior research, this multicenter, randomized, controlled clinical trial investigates the efficacy of SLLXG combined with NAC in patients with TNBC. The study analyzes the impact of SLLXG on the therapeutic response to NAC for TNBC, evaluates its potential synergistic effects with NAC, and identifies the patient population likely to benefit from this combination therapy. Develop a standardized treatment protocol with broad applicability to facilitate clinical implementation.

## Methods

### Study Design and Setting

This is a randomized, double-blind, placebo-controlled, multicenter clinical trial. This protocol complies with the SPIRIT (Standard Protocol Items: Recommendations for Interventional Trials) guidelines (Checklist 1) [22]. The study will be conducted at Longhua Hospital Affiliated to Shanghai University of Traditional Chinese Medicine, China Welfare Foundation International Peace Maternal and Child Health Hospital, Fudan University Shanghai Cancer Center, Ruijin Hospital Affiliated to Shanghai Jiao Tong University School of Medicine, Fudan University Shanghai Women’s Hospital, Baoshan Hospital of Integrated Traditional Chinese and Western Medicine in Shanghai, and Shanxi Cancer Hospital.

**Figure 1.**
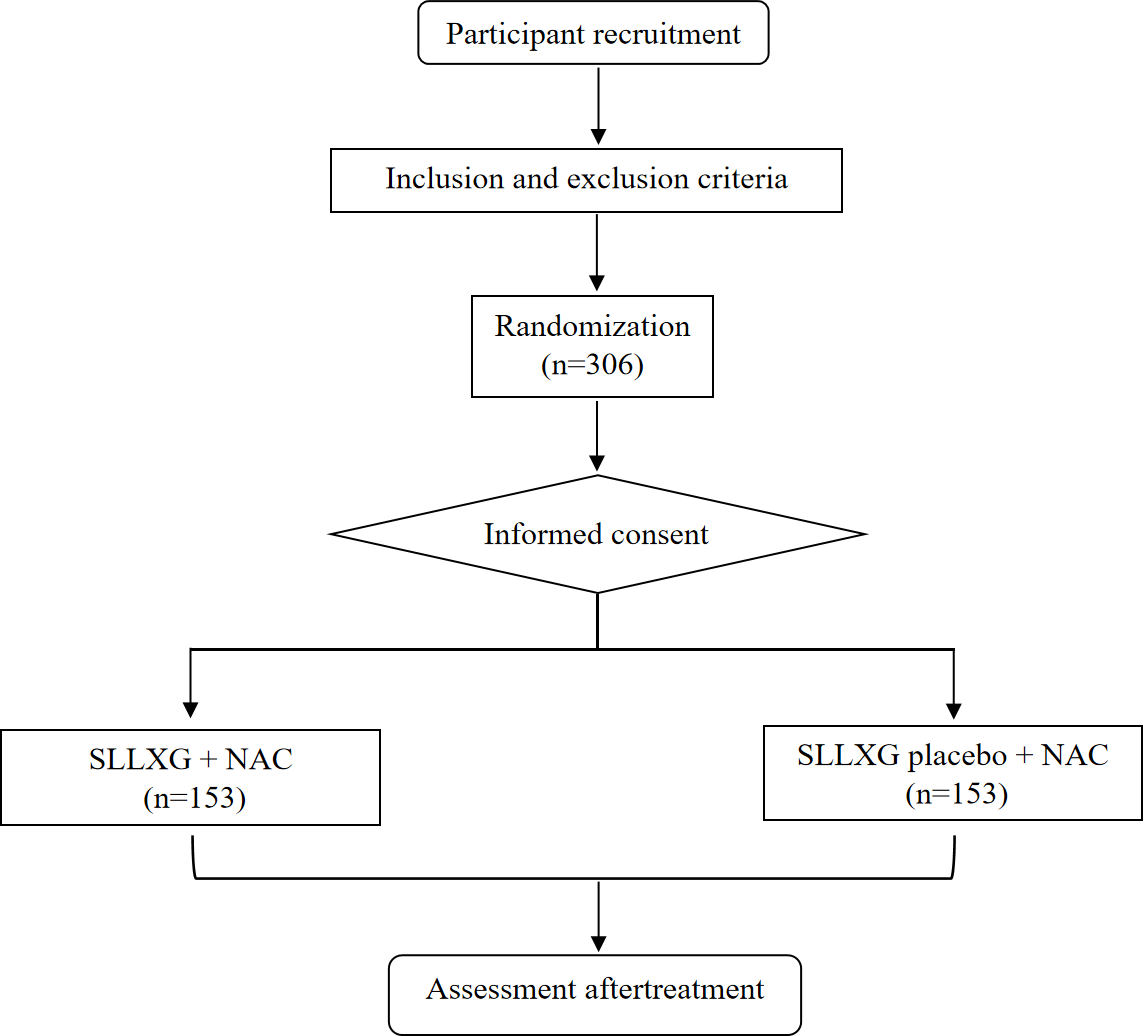
Flowchart of the research procedure. NAC: neoadjuvant chemotherapy; SLLXG: Shen-Ling-Lian-Xia Granules.

Sample size calculation was performed based on a test for superiority of 2 independent proportions. According to previous literature [23] and pilot data from the Breast Surgery Research Team at Longhua Hospital, the expected pCR rate in the control group (NAC+ placebo) is estimated at 45% (specifically for stage II-III TNBC). Based on our preliminary clinical observations, the pCR rate was observed to be 65% in patients receiving the combination therapy. Therefore, we anticipate that the combination with SLLXG will increase the pCR rate to 65% in the experimental group. Assuming a superiority margin of 3.5%, a 2-sided significance level (α) of .05, and a statistical power (1-β) of 80%, with a 1:1 allocation ratio between the two groups, the calculated sample size is 122 patients per arm. Allowing for a 10% dropout rate, a total of 306 participants (153 per group) will be recruited. The study flow is presented in Figure 1.

### Study Populations

To ensure the reliability of the study results and the safety of the participants, this study has established clear inclusion and exclusion criteria. Only participants who meet all the inclusion criteria and do not meet any of the exclusion criteria will be eligible to participate in this study. The specific inclusion and exclusion criteria for study enrollment are listed in Textbox 1.

Textbox 1. **Inclusion and exclusion criteria for enrollment into the trial.**

Inclusion criteria

- Female patients aged 18 to 75 years;
- Patients with a primary diagnosis of stage II-III triple-negative breast cancer confirmed by pathology and immunohistochemistry [24];
- Patients scheduled to receive anthracycline-based chemotherapy combined with cyclophosphamide followed by taxane-based neoadjuvant chemotherapy;
- At least one clearly measurable tumor lesion according to Response Evaluation Criteria in Solid Tumors 1.1 (RECIST) criteria for solid tumors;
- Eastern Cooperative Oncology Group (ECOG) performance status of 0-1;
- Expected survival of 6 months or longer;
- Informed consent obtained, with willingness to participate in long-term follow-up.

Exclusion criteria

- Patients with concurrent primary malignancies;
- Patients with bilateral breast cancer;
- Pregnant or lactating women;
- Patients with severe cardiac, cerebral, hepatic, renal, hematopoietic system disorders, psychiatric conditions, or significant organ dysfunction;
- Individuals who have received any form of antitumor therapy (including radiotherapy, chemotherapy, or traditional Chinese medicine [TCM]) within the past month;
- Individuals who have received TCM treatment within the past 3 months;
- Participants with a history of alcohol abuse or drug dependence; Participants enrolled in other clinical studies.

### Randomization and Blinding

To ensure a diverse and representative study population, this trial implements a multicenter competitive recruitment strategy across various clinical sites in different geographic regions. Dedicated recruitment coordinators at each center will facilitate patient identification and enrollment through hospital referral networks to ensure the sample reflects a broad range of socioeconomic and demographic backgrounds. This study uses a centralized randomization method with age as a stratification factor, using competitive enrollment across centers. An appropriate block size was selected, and random numbers were generated using SAS 9.4 statistical software based on a specified random seed number to achieve random assignment.

The study uses a double-blind design. Emergency letters accompanying study drug shipments may only be opened by preauthorized investigators in this study during urgent situations (eg, a subject experiences a serious adverse event [AEs] or drug allergy) where immediate knowledge of the subject’s treatment group is required. This constitutes emergency unblinding. Upon unblinding, the subject will be discontinued from the study and treated as a withdrawal case. The monitor will be notified of the outcome. The researcher who opened the emergency letter must document the reason for emergency unblinding, the date, and sign the letter. Detailed documentation must also be recorded in the case report form.

Additionally, the study uses a 2-stage unblinding approach. After all data are entered into the database and undergo data verification, quality control checks, and a blinding review to confirm the final statistical analysis plan, the database will be locked. The first unblinding will occur at this stage, conducted jointly by the Principal Investigator and the statistician. This involves revealing the group assignments (Group A or Group B) corresponding to each drug code to enable postgrouping statistical analysis of all data. Following analysis completion, the PI performs the second unblinding during the clinical research summary meeting, disclosing the drugs corresponding to Group A and Group B. All unblinding procedures must be documented.

### Interventions

Participants will be randomly assigned in a 1:1 ratio to one of two groups: the experimental group will receive standard NAC plus SLLXG, while the control group will receive standard NAC plus SLLXG placebo. SLLXG is composed of nine medicinal herbs: *Codonopsis Pilosula* (Dang Shen, 12g), Curcuma Zedoaria (E Zhu, 30g), Atractylodes Macrocephala (Bai Zhu, 12g), Poria Cocos (Fu Ling, 12g), Salvia Chinensis (Shi Jian Chuan, 30g), Epimedium Brevicornu (Yin Yang Huo, 15g), Solanum Nigrum (Long Kui, 30g), Scutellaria Barbata (Ban Zhi Lian, 30g), and Prunella Vulgaris (Xia Ku Cao, 9g). The granules are manufactured by Longhua Hospital Pharmacy following Good Manufacturing Practice standards. SLLXG is a registered hospital preparation approved by the Shanghai Municipal Drug Administration (Registration No. Z20230017000). To ensure the stability and controllability of the drug quality, chemical fingerprinting is performed for every batch to ensure consistency of key bioactive compounds. Recognizing that SLLXG possesses a distinct herbal odor and bitter taste, rigorous measures were taken to ensure successful blinding. To mimic the sensory characteristics of SLLXG, the placebo granules are composed of starch and food-grade colorants, containing a subtherapeutic dose (10%) of the herbal extract and a standardized food-grade herbal flavoring agent solely to replicate the specific bitterness and pungent odor of the active formula. Pretrial sensory consistency tests were conducted with independent assessors to confirm that the placebo and SLLXG were indistinguishable in appearance, scent, and flavor. Both SLLXG and the placebo will be administered orally at a dose of one sachet, twice daily. The treatment will commence on the first day of chemotherapy and continue throughout the entire NAC period.

To mitigate the risk of poor compliance during the extended treatment period, several measures will be implemented. Patients will be required to maintain a daily medication diary and return all used and unused sachets at each clinical visit for pill counting. Furthermore, dedicated research assistants will conduct bi-weekly follow-up calls or WeChat interactions to provide psychological support, monitor AEs, and reinforce the importance of adherence. These measures are designed to minimize dropouts and ensure the collection of high-quality data for the subsequent per-protocol analysis. All participants will be assigned, at the investigator’s discretion and in line with clinical guidelines, to receive one of the standard anthracycline- and taxane-based NAC regimens [25]. The specific treatment regimens are presented in Table 1.

**Table 1.** The specific treatment regimens for neoadjuvant chemotherapy.

Regimen	Drug	Dose (mg/m² iv^a^)	Administration time^b^	Administration schedule	Cycles	Total treatment duration
EC-T^c^						24 weeks
	Epirubicin and cyclophosphamide	75‐100; 600	D1	Every 21 days	4	
	Docetaxel	80‐100	D1	Every 21 days	4	
EC-wP^d^						24 weeks
	Epirubicin and cyclophosphamide	75‐100; 600	D1	Every 21 days	4	
	Paclitaxel	80‐100	D1; D8; D15	Every 21 days	4	
ddEC-ddP^e^ (G-CSF^f^ supported)						16 weeks
	Epirubicin and cyclophosphamide	75‐100; 600	D1	Every 14 days	4	
	Docetaxel	175	D1	Every 14 days	4	
ddEC-wP^g^						20 weeks
	Epirubicin and cyclophosphamide	75‐100; 600	D1	Every 14 days	4	
	Paclitaxel	80‐100	D1; D8; D15	Every 21 days	4	

a iv: intravenous.

b D1, D8, and D15 refer to days 1, 8, and 15 of each treatment cycle.

c EC-T: epirubicin and cyclophosphamide followed by taxane.

d EC-wP: epirubicin and cyclophosphamide followed by paclitaxel.

e ddEC-ddP: dose-dense epirubicin and cyclophosphamide followed by dose-dense docetaxel.

f G-CSF: granulocyte colony-stimulating factor.

g ddEC-wP: dose-dense epirubicin and cyclophosphamide followed by paclitaxel.

The use of any other Chinese or Western medicines for antitumor purposes during the treatment period is prohibited. Concomitant medications required for other conditions must be recorded in detail in the case report forms. Drug compliance will be assessed by counting returned sachets at each visit. Compliance rate = (Actual amount taken / Prescribed amount) × 100%.

### Outcomes

#### Primary Outcome

The primary outcome of this study is the pCR rate [26], defined as the absence of invasive carcinoma in the primary breast tumor and negative regional lymph nodes (ypT0/Tis, ypN0) following NAC. This pathological assessment is determined through meticulous histological examination of surgically resected tumor specimens. The complete list of primary and secondary outcomes, including their assessment tools and time points, is summarized in Table 2.

**Table 2.** Schedule of assessments.

Period	Screening period	Treatment period (SLLXG^a^/SLLXG placebo treatment)				
		NAC^b^ C0^c^	NAC C2	NAC C4	NAC C6	NAC C8
Visit	Visit 1	Visit 2	Visit 3	Visit 4	Visit 5	Visit 6
Sign informed consent	**√**					
Basic information	**√**					
Medical history	**√**					
Present illness	**√**					
Pretreatment assessment	**√**					
Item						
pCR^d^						**√**
ORR^e^			**√**	**√**	**√**	**√**
CBR^f^			**√**	**√**	**√**	**√**
MP^g^						**√**
RCB^h^						**√**
sTILs^i^						**√**
PBL^j^		**√**	**√**	**√**	**√**	**√**
EORTC QLQ-C30^k^		**√**	**√**	**√**	**√**	**√**
TCM^l^ safety evaluation			**√**	**√**	**√**	**√**
Concomitant medication	**√**	**√**	**√**	**√**	**√**	**√**
Record AEs^m^			**√**	**√**	**√**	**√**
PI^n^ review	**√**	**√**	**√**	**√**	**√**	**√**

a SLLXG: Shen-Ling-Lian-xia Granules.

b NAC: neoadjuvant chemotherapy.

c C0: cycle number of neoadjuvant chemotherapy.

d pCR: pathological complete response.

e ORR: objective response rate.

f CBR: clinical benefit rate.

g MP: Miller-Payne.

h RCB: residual cancer burden.

i sTILs: stromal tumor-infiltrating lymphocytes.

j PBL: peripheral blood lymphocyte level.

k EORTC QLQ-C30: European Organisation for Research and Treatment of Cancer Quality of Life Questionnaire-Core 30.

l TCM: traditional Chinese medicine.

m AEs: adverse events.

n PI: principal investigator.

#### Secondary Outcomes

In tumor treatment assessments, ORR and CBR are 2 pivotal metrics [27,28]. The ORR refers to the proportion of patients who achieve a complete response or partial response as determined by the Response Evaluation Criteria in Solid Tumors (RECIST) version 1.1 [29], based on serial breast magnetic resonance imaging assessments conducted throughout the course of NAC. The CBR, on the other hand, is defined as the proportion of patients who attain a complete response, partial response, or stable disease by the end of NAC, again in accordance with RECIST 1.1 criteria. Together, these metrics provide crucial insights into the effectiveness of the treatment.

Pathological assessments play a crucial role in evaluating the effectiveness of treatment. The Miller-Payne (MP) Grade [30], ranging from 1 to 5, is used to evaluate the surgical specimen, assessing the reduction in tumor cellularity compared to the baseline core biopsy. Additionally, the residual cancer burden (RCB) [31] index is calculated using an online RCB calculator from the MD Anderson Cancer Center. This calculation integrates the dimensions of the primary tumor, its cellularity, and the status of the lymph nodes. Patients are then categorized into one of four groups: RCB-0, indicating pCR, RCB-I, RCB-II, and RCB-III, which reflect varying degrees of residual disease. These assessments provide a detailed understanding of the treatment’s impact on the tumor.

Immune biomarkers play a significant role in understanding the immune response to cancer. In this context, the proportions and absolute counts of specific peripheral blood lymphocyte subsets, such as CD3^+^CD8^+^ T lymphocytes and CD3^-^CD16^+^CD56^+^ natural killer cells, will be determined using flow cytometry at designated time points [32]. Additionally, the tumor immune microenvironment will be characterized by quantifying the density of sTILs in the postoperative surgical specimen, expressed as a percentage of the stromal area [33,34]. The infiltration of CD8^+^ T lymphocytes and natural killer cells within the tumor stroma will also be assessed through immunohistochemistry. These assessments provide valuable insights into the immune landscape of the tumor and its potential impact on treatment outcomes.

Patient-Reported Outcomes are essential for assessing the impact of treatments on patients’ health and well-being. Health-Related Quality of Life is a key Outcome that will be measured using the European Organization for Research and Treatment of Cancer Quality of Life Questionnaire Core 30 [35] (EORTC QLQ-C30; version 3.0). This questionnaire includes five functional scales (eg, physical, role, cognitive, emotional, and social functioning), three symptom scales (eg, fatigue, pain, and nausea and vomiting), and a global health status/QoL scale. Scores from these scales will be calculated and compared across different groups to evaluate how treatments affect patients’ overall QoL and specific aspects of their health and well-being [36]. This comprehensive assessment helps to understand the broader implications of treatment beyond just tumor response, highlighting the importance of considering patients’ perspectives in clinical research. The complete list of primary and secondary outcomes, including their assessment tools and time points, is summarized in Table 1.

#### Safety Outcomes

Safety outcomes are a critical aspect of clinical trials, ensuring that the study interventions are well-tolerated by participants. The incidence and severity of AEs will be meticulously recorded and graded using the National Cancer Institute Common Terminology Criteria for Adverse Events [37]. This systematic approach allows for a standardized assessment of AEs and their potential relationship to the study treatments. Additionally, laboratory safety parameters will be closely monitored to detect any changes that may indicate treatment-related toxicity. These parameters include complete blood count, liver function tests (such as serum total bilirubin, alanine aminotransferase, and aspartate aminotransferase), and renal function tests (blood urea nitrogen and creatinine). By vigilantly tracking these measures, the study aims to safeguard participant health and provide a comprehensive evaluation of the safety profile of the interventions under investigation.

### Statistical Analysis

In this study, statistical analyses will be 2-sided at a 0.05 significance level, using SPSS software (version 25.0; IBM Corp.). The Full Analysis Set (FAS), following the intention-to-treat principle [38], will include all participants who received at least one dose of the intervention for primary efficacy analysis. The Per-Protocol Set (PPS) will support efficacy analysis, including only those without major protocol violations. The Safety Set (SS) will involve all participants who received the intervention and had at least one postbaseline safety assessment. Missing data for the primary outcome will be handled using Multiple Imputation or the Last Observation Carried Forward method.

The primary outcome is the pCR rate, assessed as ypT0/Tis and ypN0 postsurgery. To evaluate the effectiveness of SLLXG, the primary endpoint (pCR rate) will be compared between the NAC plus SLLXG group and the NAC plus placebo group using a logistic regression model, with the treatment group as the main independent variable and adjusting for the stratification factor (age) in the FAS. The study aims to test the hypothesis of superiority. Superiority will be demonstrated if the lower bound of the 2-sided 95% CI for the difference in pCR rates (Experimental minus Control) is greater than 0, with a 2-sided *P* value of <.05. To explore potential synergistic effects, interaction terms between SLLXG and baseline chemotherapy response indicators will be included in the model. Furthermore, to identify the best candidates for the combination treatment, a subgroup analysis and a multivariable logistic regression will be performed, incorporating covariates of interest such as age, clinical stage, and sTILs to adjust for potential confounding factors. A sensitivity analysis in the PPS will validate these findings.

An interim analysis is planned when approximately 50% of the planned sample size (n=154; 77 per group) has completed the primary outcome assessment. To maintain the overall Type I error rate, the O’Brien-Fleming alpha-spending function will be used [39], with the significance level for the interim analysis set at 0.003 and the final analysis at 0.048. This analysis will be conducted by an independent Data Monitoring Committee blinded to the investigation team. The trial may be recommended for early termination if the primary endpoint demonstrates overwhelming efficacy or futility based on the prespecified boundaries.

Secondary outcomes will be analyzed with statistical measures interpreted descriptively. To control the type I error rate across multiple secondary endpoints, the Bonferroni correction will be applied. For binary outcomes like ORR and CBR, chi-square or Fisher exact tests will be used, presenting rates and between-group differences with 95% CIs. Ordinal categorical outcomes such as MP Grade and RCB Class will be compared using the Mann-Whitney *U* test. Continuous outcomes, including laboratory values and EORTC QLQ-C30 scores for quality of life, will be assessed with appropriate parametric or nonparametric tests. Biomarker levels will be treated as continuous variables, and their associations with pCR status may be explored to generate hypotheses for future research.

### Data Management

To preserve clinical trial data integrity, a dedicated research medical record is designed for this study, serving as the source document for outpatient participants and complementing inpatient records. Data recording must be timely, complete, accurate, and verifiable, with any corrections clearly annotated and initialed by the researcher. Original lab reports should be attached to the corresponding records, and results entered into the Laboratory Results Report Form. Following each participant’s treatment cycle, researchers must submit the Research Medical Records and Informed Consent forms to the principal investigator for review within three working days.

### Ethical Considerations

This study has been approved by the Medical Ethics Committee of Longhua Hospital, Shanghai University of Traditional Chinese Medicine (approval number: 2025LCSY210; Multimedia Appendix 1). Informed consent will be obtained from all participants, who will be fully informed about the study’s details, potential risks and benefits, and their right to withdraw at any time. Prior to recruitment, patient representatives reviewed the informed consent materials to ensure clarity and readability. Additionally, stringent measures will be in place to protect the privacy of participants, ensuring that all personal data are handled confidentially and with the utmost respect for individual rights. Any substantial modifications to the protocol will be formally submitted to the Ethics Committee for approval and communicated to relevant parties accordingly. Medical treatment for any study-related injuries will be provided by the hospital, and participants will receive appropriate compensation in accordance with national regulations. Posttrial care and clinical guidance will be available to all participants after study completion.

## Results

The trial was formally funded in January 2025 (Multimedia Appendix 2). Following ethical approval in October 2025, the study is currently in the prerecruitment phase. Patient recruitment is scheduled to commence in February 2026 and is expected to conclude by June 2027, with a total target enrollment of 306 patients. Data collection for all primary and secondary outcomes is scheduled to be completed by December 2027. We plan to perform the final statistical analysis in Winter 2027, and the findings are expected to be submitted for publication in Spring 2028. To date, no deviations from the original protocol have occurred.

## Discussion

### Overview

Breast cancer has become the leading cause of cancer-related deaths among women aged 20 to 39 years [40]. Among these, TNBC is highly invasive, prone to metastasis, and associated with poor prognosis, presenting significant challenges in clinical management [41,42]. Due to its unique molecular phenotype, TNBC is insensitive to endocrine therapy or molecularly targeted therapy. The efficacy of routine adjuvant chemotherapy after surgery is poor, often leading to tumor recurrence and metastasis [43,44]. In the field of NAC, pCR is an internationally recognized key surrogate endpoint closely linked to long-term survival benefits [45,46]. Therefore, improving the pCR rate of NAC is crucial for enhancing the long-term prognosis of patients with TNBC.

Previous studies have demonstrated that SLLXG exhibits potent antitumor and metastasis-inhibiting effects [12-15]. Building on this evidence, this protocol proposes an innovative integrated therapy strategy that combines TCM with Western medicine: the addition of SLLXG to standard NAC for TNBC.

In addition to the primary endpoint of pCR rate, this study will systematically investigate the compound’s potential immunomodulatory mechanisms by examining biomarkers such as sTILs and peripheral blood lymphocyte subsets [47,48]. This is intended to provide a scientific basis for its clinical efficacy.

A pivotal milestone for SLLXG was the formalization of its technology transfer project through the Shanghai Technology Exchange. This achievement validates the formulation’s stability and readiness for advanced development. Consequently, our trial serves as a cornerstone, aiming to generate the high-quality evidence necessary to support its transition from a hospital preparation to a standardized pharmaceutical product for broader clinical benefit.

### Strengths

The multicenter design used in this study enhances subject enrollment speed, increases sample representativeness, and lends greater generalizability to the findings. Randomization, double-blinding, and placebo control constitute the gold standard for efficacy validation, minimizing bias to the greatest extent possible. The inclusion of MP grading, RCB, and quality-of-life scales establishes a multidimensional efficacy evaluation system, enabling a more comprehensive assessment of the therapeutic value of SLLXG.

### Limitations

Several limitations of this study should be acknowledged. First, despite standardized training, heterogeneity across centers may arise from variations in specific operational details and subjective assessments. Second, the rigorous eligibility criteria and the specific focus on TNBC may pose challenges to the rate of patient recruitment. Third, the extended duration of neoadjuvant treatment may impact long-term patient compliance, potentially affecting the final per-protocol analysis. Finally, a specific challenge inherent to TCM trials involves the blinding procedure. Although the placebo contains 10% of the active ingredients to mimic the sensory properties, the distinctive taste and aroma of the herbal compound may still pose a risk of unblinding. This sensory difference could potentially influence patient adherence or subject reporting, representing an unavoidable limitation in this study design.

### Prospects

Based on this research platform, future studies can conduct long-term follow-up of this cohort to evaluate the impact on long-term endpoints such as disease-free survival and OS. Concurrently, in-depth transcriptomic or proteomic analyses can be performed using biological specimens collected from this trial to explore potential therapeutic efficacy biomarkers, thereby advancing the development of personalized treatment.

### Conclusions

This study is a multicenter, randomized, double-blind, placebo-controlled clinical trial designed to systematically evaluate the efficacy and safety of the in-house preparation SLLXG combined with standard NAC for TNBC. The primary objective is to determine whether this combination therapy can significantly improve the pCR rate. The implementation of this study is expected to provide high-level evidence-based medical evidence integrating Chinese and Western medicine for the treatment of TNBC.

## Supplementary material

10.2196/91475 Multimedia Appendix 1 Ethics approval.

10.2196/91475 Multimedia Appendix 2 Grant certificate.

10.2196/91475 Checklist 1 SPIRIT 2025 checklist.

## Abbreviations

AE: adverse events
CBR: clinical benefit rate
EORTC QLQ-C30: European Organisation for Research and Treatment of Cancer Quality of Life Questionnaire-Core 30
FAS: Full Analysis Set
HPLC-MS/MS: high-performance liquid chromatography–tandem mass spectrometry
HR: hazard ratio
MP: Miller-Payne
NAC: neoadjuvant chemotherapy
ORR: objective response rate
OS: overall survival
pCR: pathological complete response
PPS: Per-Protocol Set
RCB: residual cancer burden
RECIST: Response Evaluation Criteria in Solid Tumors
SLLXG: Shen-Ling-Lian-Xia Granules
SPIRIT: Standard Protocol Items: Recommendations for Interventional Trials
SS: Safety Set
sTILs: stromal tumor-infiltrating lymphocytes
SYF: Sanyin Fang
TCM: traditional Chinese medicine
TNBC: triple-negative breast cancer
