# Supplementary material for: Efficacy and Safety of Shen-Ling-Lian-Xia Granule Combined With Neoadjuvant Chemotherapy in Patients With Triple-Negative Breast Cancer: Protocol for a Randomized, Double-Blind, Multicenter Clinical Trial
Source: JMIR Res Protoc. 2026 May 11;15:e91475. doi: 10.2196/91475 (PMC13223353; doi:10.2196/91475)
Supplement: Multimedia Appendix 1 [file resprot-v15-e91475-s001.pdf]

上海中医药大学附属龙华医院医学伦理委员会批件

伦理审议批件号：上海中医药大学附属龙华医院医学伦理委员会 2025LCSY210 号

研究名称	参苓莲夏颗粒联合新辅助化疗治疗三阴性乳腺癌的多中心随机对照研究	项目批准文号	上海市中医药管理局 GFB2504
申办单位	上海中医药大学附属龙华医院	研究方法	多中心、随机、双盲对照研究
审查类别	初始审查	审查方式	会议审查
研究单位及主要研究者	上海中医药大学附属龙华医院（组长单位） 刘 胜<br>中国福利会国际和平妇幼保健院 袁 帅<br>复旦大学附属肿瘤医院 邵志敏<br>上海交通大学医学院附属瑞金医院 陈小松<br>复旦大学附属妇产科医院 吴克瑾<br>上海市宝山区中西医结合医院 李 甜<br>山西省肿瘤医院 杨 瑞		
伦理委员会审议成员	陈晓云、王琛、周文琴、杨铭、陈文连、李久辉、吕刚、徐芝兰		
伦理委员会地址	上海市徐汇区宛平南路 725 号		
审议时间	2025-10-30		
审议结论	根据 2020 年颁布实施的《药物临床试验质量管理规范》、2022 年颁布实施的《医疗器械临床试验质量管理规范》、2010 年颁布实施的《药物临床试验伦理审查工作指导原则》、2023 年颁布实施的《涉及人的生命科学和医学研究伦理审查办法》以及《赫尔辛基宣言》，本伦理委员会审阅并讨论了下列有关材料：<br>1. 试验方案（版本号：1.1；版本日期：2025.8.20）<br>2. 研究者手册（版本号：1.1；版本日期：2025.8.20）<br>3. 知情同意书（版本号：1.1；版本日期：2025.8.20）<br>4. 研究病历（版本号：1.1；版本日期：2025.8.20）<br>5. 招募广告（版本号：1.1；版本日期：2025.8.20，海报招募）<br>6. 主要研究者简历及参研研究者履历表<br>7. 试验药物生产单位资质<br>8. 试验药物说明书<br>9. 项目任务书<br>本伦理委员会经审查同意你们自即日起开展该临床试验；并要求：<br>上述资料未经本委员会批准，不得作任何修改；试验过程中如发生严重不良事件，应立即（24 小时内）报告本委员会；<br>如临床试验方案、知情同意书及研究者有任何更改，应及时通知伦理委员会，得到重新批准；<br>请根据跟踪审查频率，提前 1 个月递交跟踪审查报告；如出现违背方案的情况，请递交违背方案报告；研究结束后 1 个月内递交研究结题报告。		
副主任委员签字	陈晓云（ ）		
批件有效期	2025 年 10 月 30 日-2026 年 10 月 29 日(跟踪审查频率 12 个月)	联系电话	021-64385700-11318
备注	请纳入第一例研究参与者之前递交合格的试验药物质检报告至伦理委员会。		

上海中医药大学附属龙华医院
医学伦理委员会（盖章）
2025-10-30
