# Supplementary material for: Efficacy and Safety of Shen-Ling-Lian-Xia Granule Combined With Neoadjuvant Chemotherapy in Patients With Triple-Negative Breast Cancer: Protocol for a Randomized, Double-Blind, Multicenter Clinical Trial
Source: JMIR Res Protoc. 2026 May 11;15:e91475. doi: 10.2196/91475 (PMC13223353; doi:10.2196/91475)
Supplement: Multimedia Appendix 2 [file resprot-v15-e91475-s002.pdf]

附件：

上海市进一步加快中医药传承创新发展三年行动计划（2025年-2027年）

项 目 建 设 任 务 书

项目编号 1-1-1

项目名称 上海市进一步加快中医药传承创新发展三年行动计划（2025年-2027年）（国家医学中心（中医类）建设）

项目牵头人 陈跃来

项目承担单位 上海中医药大学附属龙华医院 （盖章）

项目起止年月 2025年1月至 2027年12月

上海市卫生健康委员会

上海市中医药管理局

二〇二五年制

（5）	人员费用(含专家咨询费和劳务费)	17.40	17.40	5.73	11.55	0.12	/	/	/	/
（6）	其他(患者交补)	11.86	11.86	3.00	8.86	0.00	/	/	/	/
**3**	**三阴性乳腺癌，包括参苓莲夏颗粒联合新辅助化疗治疗三阴性乳腺癌的多中心随机对照研究**	**162.60**	**162.60**	**31.11**	**83.90**	**47.59**	/	/	/	/
（1）	科学研究费用	131.80	131.80	21.18	73.68	36.94	/	/	/	/
（2）	成果表达相关费用	5.60	5.60	1.53	1.82	2.25	/	/	/	/
（3）	会议和差旅费用	9.30	9.30	3.10	3.10	3.10	/	/	/	/
（4）	人员费用(含专家咨询费和劳务费)	15.90	15.90	5.30	5.30	5.30	/	/	/	/

十一、项目协议

甲方：上海市中医药管理局
同意该项目列为上海市进一步加快中医药传承创新发展三年行动计划（2025 年-2027 年）建设项目。<br>单位盖章：<br>年 月 日
**乙方：项目建设单位**
本单位承诺按照项目建设要求，根据经费预算所填写单位匹配部分，实时到账，并与市财政资助项目经费统筹管理，实物和人员经费不作为匹配经费额度。同时加强项目建设与管理，在人、财、物等方面保障项目顺利按计划完成,承担未按时完成项目的责任。<br>法定代表人签字：<br>单位盖章：<br>年 月 日
**丙方：项目牵头人**
本人承诺严格按照项目建设任务书要求按计划完项目建设任务。<br>项目牵头人签字：<br>年 月 日

上海市卫生健康委员会
上海市中医药管理局
文件

沪卫中管〔2024〕13号

上海市卫生健康委员会
上海市中医药管理局关于公布2024年度
市级医院中西医协同引导项目立项名单的通知

申康医院发展中心、有关大学，有关市级医院：

为进一步推进本市中西医协同工作，根据《关于开展2024年度综合医院中西医协同引导项目申报工作的通知》（沪卫中管〔2024〕7号），市卫生健康委、市中医药局近期组织开展了2024年度市级综合医院（含专科医院，妇幼保健院等）中西医协同引导项目申报工作。经专家评审并经市卫生健康委、市中医药局审核，确定了6个中西医协同领域及相关建设方向（见附件）。现将

立项名单予以公布，并就有关事项通知如下：

一、项目建设周期为自发文之日起两年。

二、根据确定的中西医协同领域及相关建设方向，对承担单位给予资助，鼓励承担单位给予项目经费支持。

三、市卫生健康委、市中医药局将择期组织开题汇报，指导各项目负责人按照招标文件精神，优化完善建设内容和经费用途，并填报建设任务书。

四、各办医主体和承担单位要加强对项目的指导和督促，通过建设强化中西医临床协作攻关机制，推广中西医结合医疗服务模式，提升重点领域、重点病种的中西医结合诊疗水平。

五、市卫生健康委、市中医药局将加强项目管理，并委托上海市中医优势病种管理办公室开展日常管理及验收。项目过程考核和验收结果纳入管理档案。中期考核不通过、无故中止项目或验收不通过的，将影响申报其他项目的资格。

附件：2024年度市级医院中西医协同引导项目立项名单

上海市卫生健康委员会　　　　上海市中医药管理局

2024年11月20日

（此件主动公开）

附件

2024年度市级医院中西医协同引导项目立项名单

序号	中西医协同领域	项目编号	牵头单位	项目名称	项目负责人	支持经费
1	危急重症	ZXXT-202401	上海交通大学医学院附属瑞金医院	脓毒症中西医协同诊疗新模式的创建及临床应用	瞿洪平	70万
2	神经系统	ZXXT-202402-1	复旦大学附属华山医院	经颅磁刺激联合头电针对严重意识障碍患者唤醒疗效及安全性的研究	吴雪海	45万
		ZXXT-202402-2		睡眠障碍人群的中医证候分型及失眠症状的中西医协同治疗	于　欢	25万
3	肿瘤	ZXXT-202403	复旦大学附属肿瘤医院	基于精准分型与中医证候相结合的乳腺癌中西医结合诊疗体系构建	邵志敏	50万
4	肿瘤	ZXXT-202404	复旦大学附属华东医院	直肠癌微创手术联合针药康方案改善术后直肠功能的诊疗机制和评估	韦　烨	50万
5	儿科	ZXXT-202405	上海市儿童医院	中西医协同治疗儿童原发性肾病综合征	康郁林	30万
6	呼吸系统	ZXXT-202406	上海市肺科医院	间质性肺疾病中西医联合诊疗模式探索与应用	褚海青	30万
